# The foraging distribution and habitat use of chick-rearing snow petrels from two colonies in Dronning Maud Land, Antarctica

**DOI:** 10.1007/s00227-025-04657-w

**Published:** 2025-06-05

**Authors:** Eleanor Maedhbh Honan, Ewan D. Wakefield, Richard A. Phillips, W. James Grecian, Stephanie Prince, Henri Robert, Sébastien Descamps, Anna Rix, A. Rus Hoelzel, Erin L. McClymont

**Affiliations:** 1 Department of Geography, Durham University, Durham, DH1 3LE UK; 2 Natural Environment Research Council, British Antarctic Survey, High Cross, Madingley Road, Cambridge, CB3 0ET UK; 3 International Polar Foundation, Rue des Vétérinaires, 42b/1, Brussels, 1070 Belgium; 4 Norwegian Polar Institute, Fram Centre, Tromsø, 9296 Norway; 5 Department of Biosciences, Durham University, Durham, DH1 3LE UK

**Keywords:** Procellariiformes, Fulmarine petrel, Sea ice, Habitat preference, Seabird distribution, Climate change

## Abstract

The polar sea-ice zones are highly productive and seasonal habitats that support large populations of vertebrate predators. In the Antarctic, snow petrels (*Pagodroma nivea*) are regarded as highly ice-dependant, yet knowledge of their habitat use and foraging distribution during the breeding period comes largely from ship-based observations. Snow petrels show sexual size dimorphism, and previous studies have demonstrated a degree of sexual segregation in habitat use in East Antarctica during the incubation period. Here, we characterise the movements, behaviour and habitat use of foraging snow petrels using bird movement and remotely sensed environmental data. We tracked snow petrels from two colonies in Dronning Maud Land, East Antarctica during early chick rearing in January to February 2022, a period of unusually rapid sea-ice retreat in the region. During the chick-rearing period, mean foraging range was c. 395 km and did not differ between the sexes. However, we found some evidence of differing habitat use between the sexes: males foraged more in areas of higher sea-ice concentration and over the continental shelf, while females utilized deeper waters and lower sea-ice concentrations. Sexes also diverged in their behavioural responses to both sea ice and depths, with males more likely to switch to foraging in areas of higher sea-ice concentrations than females and females more likely to switch to foraging in deeper waters than males. Although both sexes were more likely to forage at higher sea-ice concentrations, they also used areas with little or no sea ice. This contrasts with previous studies and may have been due to the unusual paucity of sea-ice cover during our tracking period.

**Supplementary Information:**

The online version contains supplementary material available at 10.1007/s00227-025-04657-w.

## Introduction

The Antarctic sea-ice zone is one of the world’s largest, most dynamic and productive marine ecosystems (Massom and Stammerjohn 2010). The cycle of advance and retreat of sea ice is a primary driver of ecosystem dynamics through its influence on primary production (Michel et al. 2019; Norkko et al. 2007; Rossi et al. 2019). It also determines access to breeding, resting and foraging grounds for air-breathing marine predators such as seabirds, cetaceans and pinnipeds. The life histories of Antarctic animals are therefore coupled with the annual cycle of the sea ice (Le Guen et al. 2018; Maccapan et al. 2023).

Since the advent of continuous satellite records in the 1970 s, Antarctic sea ice showed a trend towards a slight increase in overall extent, although there were strong regional advances and retreats (Parkinson and Cavalieri 2012). However, summer sea-ice extent fell to an unprecedented low in 2017, and following a period of apparent recovery (Eayrs et al. 2021) underwent the most rapid summer retreat on record in 2022 to a new low: in the Weddell Sea, this was 26% below the 30-year mean (Fogt et al. 2022; Turner et al. 2015, 2022). In the same year, Dronning Maud Land experienced extreme weather, with exceptionally high snowfall and windspeeds, leading to near-complete breeding failure at some seabird colonies (Descamps et al. 2023). Since 2022, further record sea ice minima have occurred (Gilbert and Holmes 2024). Further declines in sea-ice extent, together with resultant changes in primary and secondary production, are expected to have cascading effects through the food web of the Southern Ocean (Gutt et al. 2021).

The sea-ice zone is a crucial foraging habitat for Antarctic predators (Bestley et al. 2020). However, due to current gaps in our understanding of their foraging habits and distribution (Delord et al. 2016; Lowther et al. 2022) predicting the impact of sea-ice loss on predators, including threatened groups such as seabirds (Dias et al. 2019), remains a major challenge (Gutt et al. 2021). Much of our understanding of at-sea distributions of Antarctic seabirds comes from ship-based observations (Warwick-Evans et al. 2021). These can be used to assess density, distribution and behaviour but ship-based surveys tend to be relatively limited in spatial and temporal coverage, and cannot resolve many characteristics (sex, provenance, life-history stage, etc.) of the observed animals (Ainley et al. 2017). In contrast, tracking with bird-borne devices can provide insights into variation in foraging behaviour among colonies, breeding stages, ages, sexes and individuals (Ballance 2007; Phillips et al. 2017). To assess how climate change may affect pagophilic (sea-ice-associated) Antarctic seabirds, we need to understand how they exploit sea-ice habitats, and the environmental processes underlying this habitat use (Hazen et al. 2019).

The snow petrel *Pagodroma nivea* is the smallest fulmarine petrel, with a circumpolar Antarctic breeding and foraging distribution (Francis et al. 2025). Population sizes are uncertain in all regions but a minimum global total of ~ 77,400 breeding pairs was estimated by a recent review (Francis et al. 2025). Snow petrels breed in crevices under boulders or large rocks on islands or up to 400 km inland on nunataks on the Antarctic continent (Francis et al. 2025; Goldsworthy and Thomson 2000). Previous tracking of snow petrels at continental colonies indicated that they remained largely in the pack ice throughout the year (Barbraud et al. 2021; Delord et al. 2016; Viola et al. 2023). The only previous GPS-tracking study took place at Pointe Géologie (Terre Adélie) during the incubation stage, and there snow petrels foraged in areas where sea-ice concentration (hereafter SIC) ranged between 30 and 78% (Barbraud et al. 2021). Snow petrels are highly sexually dimorphic (Barbraud 2000; Barbraud et al. 1999), and males and females tracked at Terre Adélie during incubation exhibited differing habitat use with respect to depth and sea-ice concentration (Barbraud et al. 2021). It is unknown whether snow petrels use similar habitats during chick rearing, or in regions with differing sea-ice availability, and if sex-based differences in habitat use persist outside of the incubation period.

Here, we aimed to quantify foraging trip characteristics, space use and habitat use of breeding snow petrels in Dronning Maud Land, East Antarctica during chick rearing, when seasonal sea ice retreats almost to the coast. To do so, we tracked birds from two colonies, approximately 720 km apart, in the austral summer of 2021/22. We tested for differences between sexes and colonies in foraging trip characteristics and habitat use. Our study coincided with a period of anomalously extreme weather and record minimum summer sea-ice extent in the region (Descamps et al. 2023; Turner et al. 2022), and was later in the seasonal melt cycle of sea ice than the Barbraud et al. (2021) study. Our results are timely given the predicted increase in frequency and intensity of extreme events as a consequence of climatic change (Siegert et al. 2023).

## Materials and methods

### Study species and sites

Snow petrels lay one egg between late November and early mid-December, and hatching occurs in January after an incubation period of approximately 43 days. Chicks are brooded by both parents for 2–10 days, and then fed by both parents until fledged at c. 46 days (Marchant and Higgins 1990). Adults depart the colony in mid-March and disperse to the sea-ice zone for the non-breeding period (Delord et al. 2016; Viola et al. 2023).

**Fig. 1:**
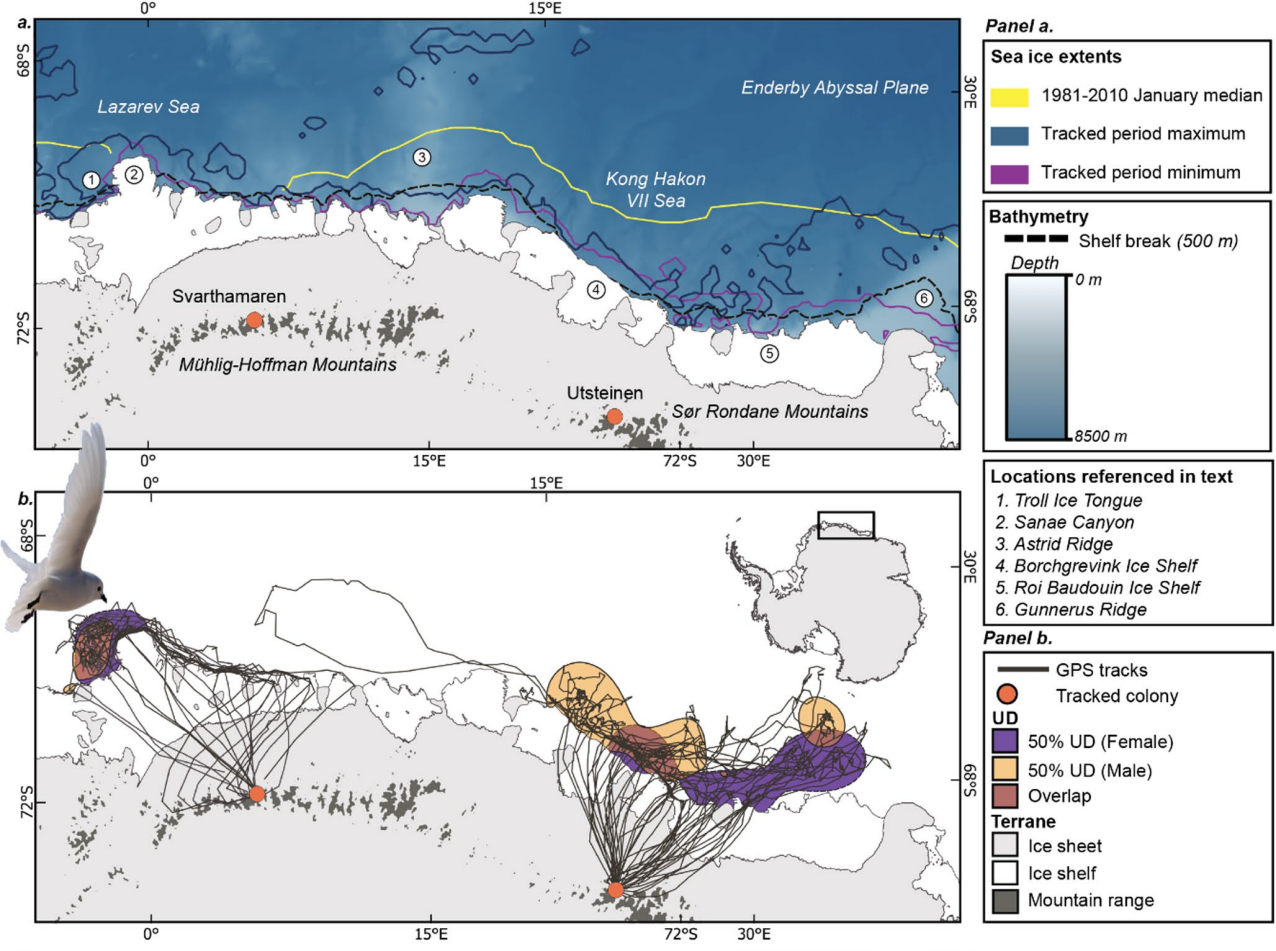
***a*** Study area, bathymetry, sea-ice edges and locations referenced in the text. ***b*** Foraging trips and core 50% utilisation distributions (UDs) of snow petrels of both sexes tracked from Svarthamaren and Utsteinen, Dronning Maud Land, Antarctica, during chick rearing in January – February 2022

Fieldwork was carried out in eastern Dronning Maud Land, Antarctica. We tracked snow petrels from Utsteinen nunatak (71°57’S, 23°21′E), at the northern edge of the Sør Rondane Mountains, between the 22nd of January and 12th of February, 2022, and Svarthamaren nunatak (71°53′S, 5°10′E) in the Mühlig-Hofmann Mountains, from the 16 th of January to the 4 th of February, 2022. The study colony at Utsteinen holds c. 60 breeding pairs (HR, unpubl data*).* Svarthamaren is an Antarctic Specially Protected Area (ASPA, No. 142) and Important Bird Area designated for both snow petrels and Antarctic petrels, with an estimated population of 2000 breeding pairs of the former (SD, unpubl data). Both colonies are c. 200 km from the coastal margin, as delimited by the edge of the Antarctic ice sheet (Fig. 1).

### GPS tracking

We captured breeding adults on the nest either by hand or with a monofilament noose attached to a 1 m pole during late incubation and chick rearing. We removed three contour feathers from the lower back or breast for molecular sexing. Each bird was weighed to ± 5 g then a NanoFix GEO remote-download GPS logger (Pathtrack Ltd., Otley, UK; 23 × 13 × 8 mm, plus 50 mm whip antenna) was attached to the base of the two innermost rectrices using Tesa tape. GPS loggers had either a battery and solar panel, or a battery only, and weighed 3.9 g (range 3.9–4.0 g) and 3.4 g (range 3.1–3.4 g), respectively. This corresponded to 0.5–0.8% of individual body mass, considerably less than the 1–3% threshold above which negative impacts on seabird breeding behaviour have been detected (Bodey et al. 2018; Phillips et al. 2003). GPS loggers were programmed to record one location every 30 min. Birds were returned to the nest after deployment and monitored for > 10 min to check they did not leave the nest crevice. Handling time was < 15 min per individual. In each colony, GPS data downloaded automatically to base stations placed near the nests. If possible, GPS loggers were retrieved after one or more foraging trips. Any loggers not retrieved would have been shed at the latest during tail moult, which occurs in the post-fledging period in March-April (Marchant and Higgins 1990). Due to the limited period for fieldwork, we were unable to test for impacts of the devices on breeding success or body condition. We recorded only three trips during incubation (all from Utsteinen) and three during brood-guard (all from Svarthamaren). Due to these small sample sizes, we excluded incubation trips and combined brood-guard and post-brood trips for comparisons between colony and sex, considering these as representative of early chick-rearing.

### Sex determination

We determined the sex of tracked birds via standard molecular analysis using primers (2550 F and 2718R) previously used for snow petrels (Fridolfsson and Ellegren 1999; Barbraud et al. 2021). We isolated total genomic DNA from body feather tips using the tissue DNA isolation protocol and reagents of the E.Z.N.A. Tissue DNA kit (Omega Bio-tek; Norcross, GA, USA), and amplified DNA using Platinum II Taq Hot-Start DNA polymerase (Invitrogen) for 50 cycles with annealing at 50 °C for 25 s and extension at 68 °C for 15 s. PCR products were examined using 1.2% agarose gels. All PCR amplifications and gels included one known female snow petrel and a negative PCR control of water. Samples resulting in no bands were rerun using differing amounts of DNA until sex could be established definitively. Birds that produced only the CHD1Z band (approximately 650 bp) were designated as male. Birds that produced both the CHD1Z and CHD1 W band (approximately 450 bp) were designated as female, and birds that produced only the CHD1 W band or the CHD1 W band and a very faint CHD1Z band were designated as female.

### Trip metrics

All analyses were carried out in R v.4.4.3 (R Core Team 2024) and QGIS v 3.38.0 (QGIS Association 2024). We used the R packages *sf* (Pebesma et al. 2024) and *terra* (Hijmans et al. 2025) to extract environmental data and calculate metrics for each GPS location. We used generalised linear mixed effect models (GLMMs) implemented in the R package *glmmTMB* to test for differences between sexes and colonies in trip metrics and habitat use (Brooks et al. 2024). We checked conformity to model assumptions using the *“simulateResiduals”* function of the *DHARMa* R package (Hartig et al. 2024) and used the *“r.squaredGLMM”* function of the *MuMin* R package (Bartoń 2024) to generate marginal and conditional R^2^ values for all models, except those with Tweedie error distributions. Significance of all statistical tests was determined at the *p* < 0.05 level and values are reported as means ± standard errors, or medians with interquartile ranges (IQRs) in square brackets.

We divided the GPS tracks into discrete foraging trips (i.e., excursions from the colony to locations at sea and back) using the *“tripSplit”* function in the *track2 KBA* package (Beal et al. 2021), classifying trips with data streams terminating > 40 km from the colony as incomplete. We used a high-resolution vector polygon of the Antarctic ice sheet (Gerrish et al. 2024) to identify trip segments that were over the ice sheet. For all habitat analyses, only the trip locations over the sea were retained, and we discarded trips in which data coverage during the period at sea was incomplete. We defined outward and inward commuting legs of trips as those in which the bird was over the ice sheet flying from the colony to the coast and vice-versa. Using only complete trips, we tested for differences between colonies and sexes in the following trip metrics: Total distance travelled (km), maximum range (distance to furthest location reached from the colony, km), total duration (hours), total time spent at sea (i.e., excluding commuting between the colony and coast, hours), total commuting time (hours) and commuting distance (km) between the colony and coast. To test for differences in these trip metrics between colonies and sexes, we used GLMMs to model the mean trip-level value of each metric. To account for repeated observations, we included bird-level intercepts as random effects. We specified error families (Gamma or Gaussian), and link functions (log or identify) based on inspection of the data and diagnostic plots of residuals and Q-Q plots (Hartig et al. 2024).

We used the R packages *geosphere* and *circular* (Hijmans et al. 2024; Lund et al. 2024) to calculate the bearings at which tracked birds departed from and arrived at the colony. Bearings were defined as the heading from the colony to the first or last recorded location over sea ice or open water.

### Environmental data

Previous studies have shown snow petrels and their prey to be associated with particular sea-ice conditions and bathymetry (Ainley et al. 1984; Barbraud et al. 2021; Ribic et al. 2011),so we used indices that describe these features to quantify habitat use. To quantify sea-ice concentration at foraging locations, we used ASI (AMSR2/ARTIST Sea Ice algorithm) version 5.4 sea-ice concentration data on a regular 3.125 km polar stereographic grid (Spreen et al. 2008) downloaded from the Institute of Environmental Physics, University of Bremen (https://seaice.unibremen.de, downloaded May 2023). Using the gridded SIC data, we calculated the minimum distance of each GPS location to the sea-ice edge, which we nominally defined as the 15% SIC contour (Worby and Comiso 2004; Massom and Stammerjohn 2010; Windnagel et al. 2017). We specified negative distances to the sea-ice edge for locations within the pack ice (areas with > 15% sea ice), and positive distances for locations in open water (≤ 15% sea ice). Summer sea ice can recede to the coastline of eastern Dronning Maud Land (Lowther et al. 2022) and so at times, the edge of the ice sheet, which is fringed with a narrow band of fast ice, represented the sea-ice edge.

To quantify bathymetry, we used the International Bathymetric Chart of the Southern Ocean (IBSCO v2, https://ibcso.org) with a 500 × 500 m resolution (Dorschel et al. 2022, 2024). Using these data, we nominally defined the continental shelf break as the 500 m depth contour. We calculated the distance of each GPS location to this contour. Distances from the shelf break to neritic locations (i.e., landward of the shelf break) were assigned as negative, and distances to oceanic locations (i.e., seaward of the shelf break) as positive. We calculated distance from the coastline for each GPS location using a high-resolution vector polygon of the Antarctic coastline (BAS Data Catalogue) (Gerrish et al. 2024).

### Behavioural states and habitat use

We used Hidden Markov Models (HMMs, (Bennison et al. 2018; Langrock et al. 2012), fitted in the *momentuHHM* R package (McClintock and Michelot 2018, 2022) to identify putative behavioural states at bird locations based on turning angles and step lengths, and to model the effects of environmental covariates on the probability of switching between states (Grecian et al. 2018). We first removed trips where the number of recorded location points was low compared to the expected number based on the trip duration and tracking interval of 30 min, removing trips which had less than 40% of the expected location points. We then linearly interpolated trips to 30-minute intervals using the R package *adehabitatLT* (Calenge 2006). Snow petrels sometimes forage by alighting on floating ice, for example, when scavenging carrion (Ainley et al. 1984; Ridoux and Offredo 1989). However, they also rest on icebergs and floes (Ainley et al. 1993; Joiris 2018), making it difficult to distinguish these behaviours using turning angles and step lengths, especially given the relatively low temporal resolution and our small sample size. We therefore assumed that only two behavioural states could be discriminated: Transiting, with longer step lengths and persistent directionality; and non-transiting, characterised by shorter step lengths and wide dispersed turning angles, which we assume encompasses both foraging and resting.

We assumed that step lengths and turning angles followed Gamma and wrapped Cauchy distributions, respectively. The latter was centred around zero with a concentration parameter *ρ* ranging from zero when angles are uniformly dispersed to one when they are concentrated in one direction (McClintock and Michelot 2018). We chose a realistic range of initial starting parameters for step length and turning angles based on visual inspection of the data and a priori knowledge of procellariform movement (Table S1). These were then refined by refitting the model 25 times using starting parameters drawn randomly from within these ranges, and retaining the model with the lowest log-likelihood (Michelot and Langrock 2023). Taking this as the null model, we then used Akaike Information Criterion (AIC) and forward selection to determine whether the inclusion of sex and environmental covariates improved the model. Given the imbalance of trips obtained from the two study colonies, and the lack of a priori reasoning for colony of origin to influence behavioural state characteristics, we did not include colony as a covariate in the models. From a suite of biologically plausible environmental variables, we identified those which were not collinear and included only these – sea-ice concentration, distance to the sea-ice edge and depth – and their two-way interactions and second-degree polynomials. State-dependent distributions for step lengths and turning angles were modelled without covariates, assuming that within states, the distributions of step lengths and turning angles did not vary systematically with environmental conditions. We used pseudo-residual plots to check model assumptions (McClintock and Michelot 2018). Using the best-fit model and the Viterbi algorithm (Zucchini et al. 2017), we determined the most likely sequence of behavioural states for each trip, then visually checked that these were biologically realistic. To determine activity budgets, we took the trip-level distributions of time spent in each state and found the median time per state for individuals. We then tested whether the proportion of time spent in the non-transit state differed between the sexes and colonies using a GLMM with a beta regression and a logit link function, specifying bird ID as a random intercept.

We estimated Utilisation Distributions (UDs) for males and females at both colonies separately during the non-transit state using the *adehabitatHR* package, with smoothing parameters chosen using the ad hoc method *“href”* (Calenge 2006).

To assess how probability of switching between states varied with environmental conditions, we plotted transition probabilities as functions of sex and each covariate retained in the best fitting Hidden-Markov model. We classified locations with a SIC of < 15% as open water, ≥ 15% – < 80% as the marginal ice zone (MIZ), and > 80% as consolidated pack ice (Wakefield et al., 2024, Stroeve et al., 2016). For depth, we categorized locations < 500 m as continental shelf, 500–3000 m as continental slope, and > 3000 m as oceanic (Dorschel et al. 2022, 2024).

From the transition probabilities, which represent the immediate likelihood of switching between states, we calculated and plotted stationary-state probability distributions for each covariate, separately for each sex. Stationary-state probabilities represent the equilibrium probabilities of being in a given state under fixed values of a covariate in the Markov process, summarizing the long-term behaviour under specific environmental conditions (Patterson et al. 2009). For sea-ice concentration, depth was fixed at its mean value, and for depth, sea-ice concentration was fixed at its mean value.

Finally, to test whether habitat use, which we use to define the environmental conditions at locations used for putative foraging and resting behaviour, differed between colonies and sexes, we used GLMMs to model the trip-level median of each environmental covariate as a function of sex and colony for non-transit locations only. We included bird-level random intercepts and specified error families and link functions based on inspection of the data and diagnostic plots of residuals and Q-Q plots (Hartig et al. 2024).

## Results

### Foraging trip characteristics

Of 40 deployments, we obtained GPS data from 21 individuals, comprising 40 trips in total. Logger deployment durations were 25.5 to 142.6 h for complete trips, and we obtained a median of 1 trip per bird (IQR 0.6–1.3; range 1–6). Device retrieval rate was 100% at Svarthamaren (6 of 6 devices) and 41.1% at Utsteinen (15 out of 34 devices). The lower rate at Utsteinen was largely due to intense storms during late incubation (Descamps et al. 2023), causing desertion of nests by instrumented birds and many others in the colony. Mean body mass at deployment was 240 ± 27 g (range 225–290 g) at Svarthamaren, and 260 ± 26 g (205–325 g) at Utsteinen and did not differ significantly between colonies (Wilcoxon rank sum test, *W* = 69, *p-value* = 0.25) (Table S2). For both colonies, we found that direction of colony departure and arrival differed, usually resulting in clockwise foraging trips. Birds leaving the colony did so with mean departure bearings of 336.8° (*ρ* = 0.09) from Utsteinen and 312° (*ρ* = 0.14) from Svarthamaren and returned with mean arrival bearings of 20.4° (*ρ* = 0.07) to Utsteinen and 8.6° (*ρ* = 0.4) to Svarthamaren.

**Fig. 2:**
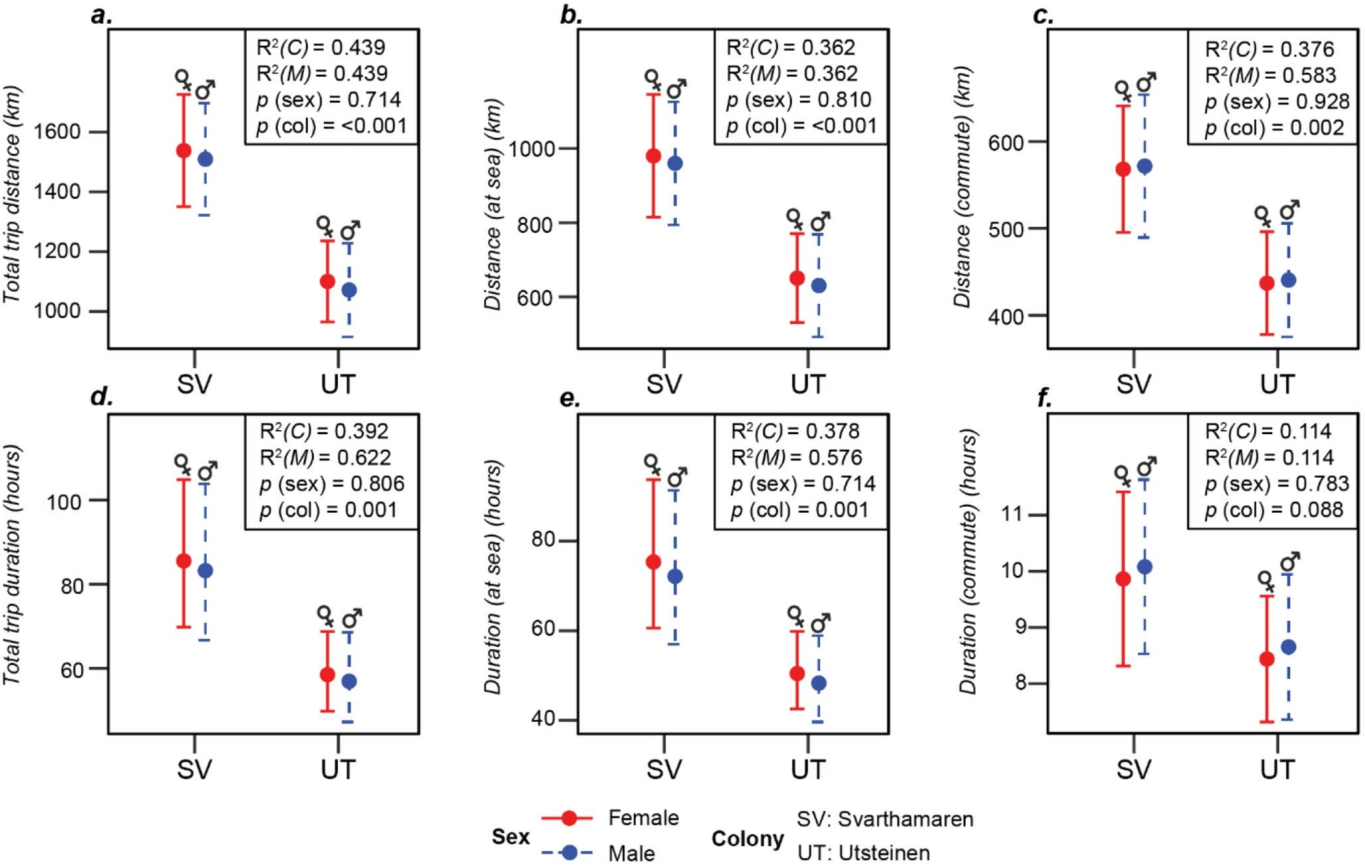
Differences in metrics describing the distance and duration of foraging trips made by snow petrels tracked from two colonies in Dronning Maud Land, Antarctica estimated using the Generalised Linear Mixed-effect Models summarised in Table 1 (for sample sizes, Tables S1 and S2). M = marginal and C = conditional R^2^ of the models

Of the 40 trips tracked, 25 were complete and therefore included in our analysis of trip metrics. This represented a sample of 14 individuals, seven females and five males from Utsteinen, and four females and two males from Svarthamaren (Tables S3 and S4). There were no significant differences between sexes in trip distance or duration (Fig. 2; Table 1). Trips of birds from Svarthamaren took longer, covered greater distances and involved more time spent at sea than those from Utsteinen (Fig. 2; Table 1).

**Table 1:** Fixed effects in generalised linear mixed-effects models of trip distance and duration metrics at non-transiting locations used by snow petrels tracked from two colonies in Dronning Maud land, Antarctica^i^

Response (units, error family, link function)	Covariate	Estimate	SE	z		*p*
Total duration *(hours*,* Gamma*,* Log)*	Intercept (females, SV)	4.448	0.104	42.89		< 0.001
	Sex (males)	−0.379	0.116	−3.28		0.806
	Colony (UT)	−0.027	0.111	−0.25		< 0.001
Duration at sea *(hours*,* Gamma*,* Log)*	Intercept (females, SV)	4.322	0.111	38.87		< 0.001
	Sex (males)	−0.043	0.119	−0.37		0.714
	Colony (UT)	−0.4	0.123	−3.26		0.001
Total commute duration *(hours*,* Gaussian*,* Identity)*	Intercept (females, SV)	9.8	0.790	12.48		< 0.001
	Sex (males)	0.2	0.785	0.276		0.783
	Colony (UT)	−1.4	0.835	−1.707		0.088
Total distance *(km*,* Gaussian*,* Identity)*	Intercept (females, SV)	1538.1	95.71	16.071		< 0.001
	Sex (males)	−28.63	95.01	−0.301		0.763
	Colony (UT)	−437.77	101.10	−4.330		< 0.001
Distance covered at sea *(km*,* Gaussian*,* Identity)*	Intercept (females, SV)	980.34	84.48	11.604		< 0.001
	Sex (males)	−20.09	89.24	−0.240		0.810
	Colony (UT)	−329.56	83.87	−3.693		< 0.001
Total commute distance (*km*,* Gaussian*,* Identity)*	Intercept (females, SV)	568.14	37.151	15.293		< 0.001
	Sex (males)	3.605	40.08	0.090		0.928
	Colony (UT)	−131.149	43.126	−3.041		0.002

^i^ Model data compromises 25 trips made by 14 snow petrels. See supplementary tables S1 and S2 for trip counts

**Fig. 3:**
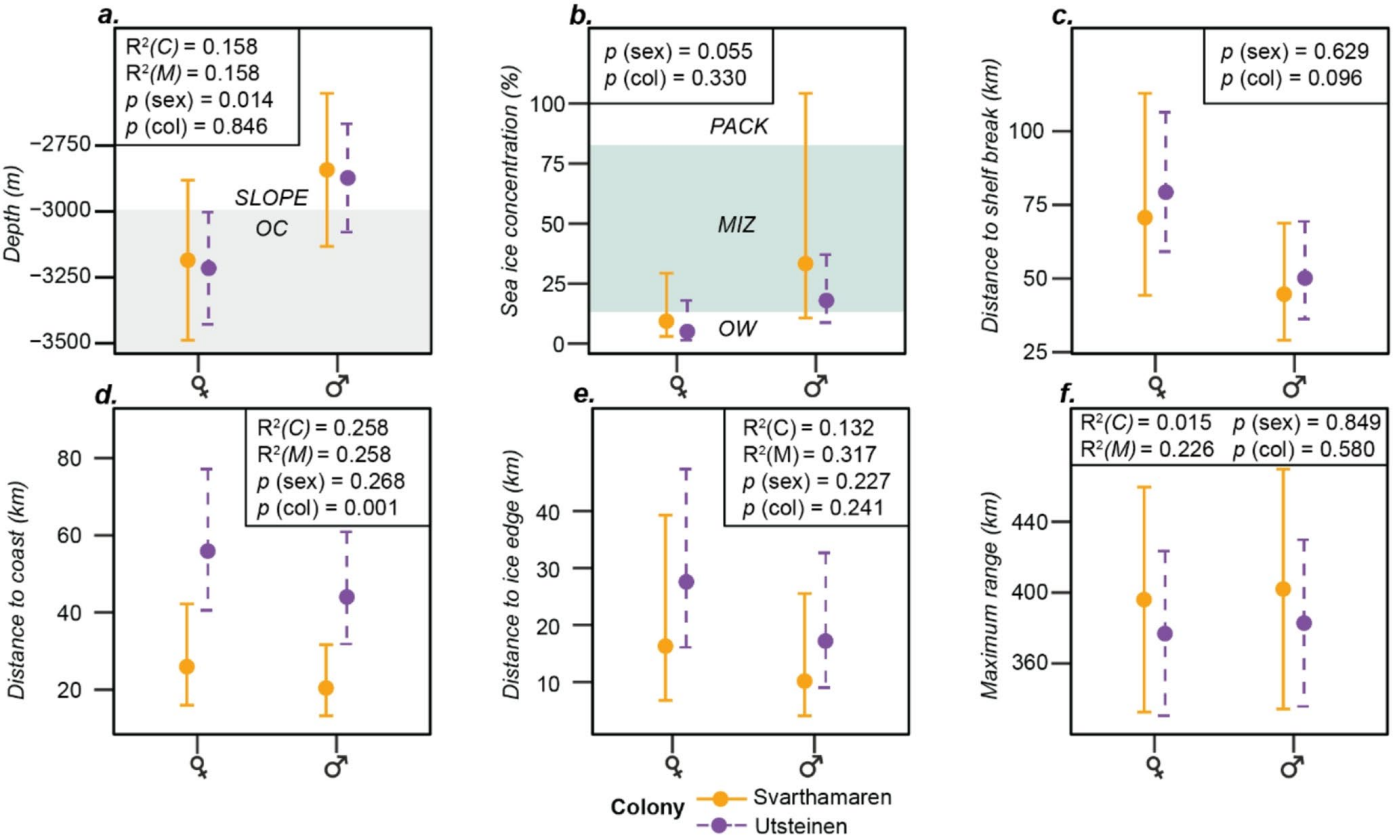
Generalised linear mixed-effects models predicted values for habitat use with respect to sex and colony. Model structures are summarised in Table 2. Model data compromises 34 trips made by 15 snow petrels. See supplementary tables S1 and S2 for details on the number of trips obtained from individual birds. Reported figures are *p* values for sex and colony comparison alongside the marginal and conditional R^2^ values (M = marginal and C = conditional). It was not possible to calculate R^2^ for Tweedie distribution models. See tables S1 and S2 for sample sizes. Slope = Continental slope. OC = Oceanic waters. Pack = Pack Ice (sea-ice concentrations > 80%). MIZ = Marginal Ice Zone (sea-ice concentrations < 80%  – > 15%). OW = Open waters (< 15% sea-ice concentration). ***(a)*** Depth ***(b)*** Sea-ice concentration ***(c)*** Distance to shelf break ***(d)*** Distance to the coast ***(e)*** Distance to the sea-ice edge ***(f)*** Maximum range

The mean maximum range of non-transit (i.e., foraging or resting) locations from the colony was 395.9 ± 32.4 km, and neither this nor mean environmental conditions at these locations differed significantly between the two colonies (Fig. 3; Table 2). The only exception was that birds from Utsteinen used locations ~ 55 km from the coast, whereas birds from Svarthamaren used locations ~ 30 km further inshore. Neither mean distance to the ice edge (16.2 ± 0.4 km) or coastline (25.9 ± 0.2 km) differed between the sexes.

**Table 2:** Fixed effects in generalised linear mixed-effects models of environmental conditions at non-transiting locations used by snow petrels tracked from two colonies in Dronning Maud land, Antarctica^i, ii^

Response (units, transformation, error family, link function)	Covariate	Estimate	SE	z	*p*
Depth *(m*,* none*,* Gaussian*,* Identity)*	Intercept (females, SV)	3185	155	−20.564	< 0.001
	Sex (males)	−342	139	2.471	0.014
	Colony (UT)	31	157	−0.195	0.846
Sea-ice concentration *(%*,* none*,* Tweedie*,* Log)*	Intercept (females, SV)	2.237	0.583	3.839	< 0.001
	Sex (males)	1.270	0.661	1.92	0.055
	Colony (UT)	−0.618	0.634	−0.974	0.330
Distance to shelf break *(km*,* +min*,* Tweedie*,* Log)*	Intercept (females, SV)	4.258	0.239	17.834	< 0.001
	Sex (males)	−0.458	0.207	−2.211	0.629
	Colony (UT)	0.115	0.239	0.483	0.095
Distance to sea-ice edge *(km*,* +min*,* Gamma*,* Log)*	Intercept (females, SV)	2.790	0.449	6.212	< 0.001
	Sex (males)	−0.473	0.391	−1.209	0.227
	Colony (UT)	0.526	0.448	1.174	0.241
Distance to coastline *(km*,* none*,* Gamma*,* Log)*	Intercept (females, SV)	3.256	0.248	13.114	< 0.001
	Sex (males)	−0.240	0.216	−1.108	0.268
	Colony (UT)	0.7679	0.245	3.141	0.002
Maximum Range *(km*,* none*,* Gaussian*,* Identity)*	Intercept (females, SV)	395.993	32.405	12.22	< 0.001
	Sex (males)	5.951	31.166	0.191	0.849
	Colony (UT)	−19.205	34.695	−0.554	0.580

^i^Model data compromises 34 trips made by 15 snow petrels. See supplementary tables S1 and S2 for trip counts

^ii^Data from both colonies were pooled for these models

### Behavioural states and habitat use

The at-sea portions of 34 trips were complete and therefore included in the behavioural HMMs and habitat-use analysis. This represented a sample of 14 individuals: seven females and four males from Utsteinen, and three females and one male from Svarthamaren. (Tables S3 and S4). The HMM distinguished one state with short to intermediate step lengths (2709 ± 2713 m) and weak directionality (*ρ* = 0.45), from another state with long step lengths (11247 ± 4809 m) and high directional persistence (*ρ* = 0.84) (Fig. S2, Table S1). Both sexes spent approximately half of their time in each of the two states, and this did not differ between colonies (Table S5).

On average, the sexes foraged or rested in areas with different depths and SICs (Fig. 3; Table 2): Females used waters ~ 3185 m deep, with SICs of 9%, whereas males used waters ~ 340 m shallower, at mean depths of 2843 m and SICs 22% greater, at mean concentrations of 33%, although the latter difference was only marginally significant (*p* = 0.054).

The core (50%) utilization distributions of both males and females from Svarthamaren was centred west of the Troll ice tongue (Fig. 1b), with most usage occurring within < 20 km of the feature. One female foraged further west, close to the coastline in the region directly over the Sanae Canyons. All chick-rearing trips from Utsteinen were within the Riiser-Larsen Sea basin, bordered by the Gunnerus Ridge to the east and the Astrid Ridge to the west (Fig. 1a, b). The 50% UDs for each sex overlapped in an area due north of the colony, with the 50% UD for males extending to the west, along the length of the Borchgrevink Ice Shelf, and for females to the east, along the coast of the Roi Baudouin Ice Shelf. During early chick-rearing, there was no overlap between the core UDs of colonies. Throughout the study, only one individual from Utsteinen crossed into the 95% UD for Svarthamaren. In that case, a female in early post-brood travelled further west, crossing the Astrid Ridge and into open water far from the sea-ice edge in the Lazarev Sea.

**Fig. 4:**
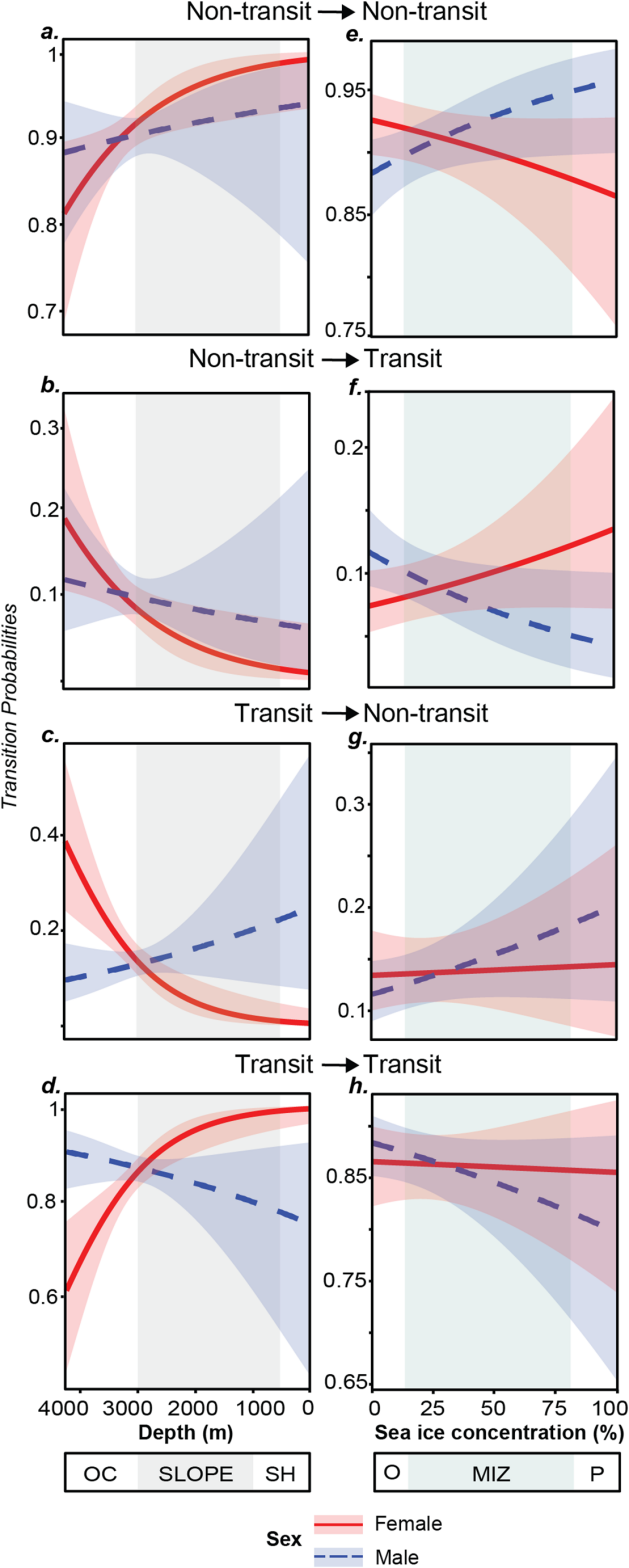
Transition probabilities for two Hidden Markov Model-estimated behaviours of snow petrels from two colonies in Dronning Maud Land for females (red lines) and males (blue dashed lines) with respect to a range of water depths (***a–d***) and sea-ice concentrations (***e–h***). Solid/dashed lines show means and shaded error bars show 95% confidence intervals. As the colony of origin did not impact our results, data from both colonies is pooled. The shaded area on panels ***a–d*** represents the continental slope and in ***e–h*** the marginal ice zone. SLOPE = Continental slope. OC = Oceanic waters. SH = Waters over the continental shelf. Pack = Pack Ice (SIC > 80%). MIZ = Marginal Ice Zone (SIC < 80% - > 15%). O = Open waters (< 15% SIC). Model data compromises 34 trips made by 15 snow petrels. See supplementary tables S1 and S2 for trip counts

**Fig. 5:**
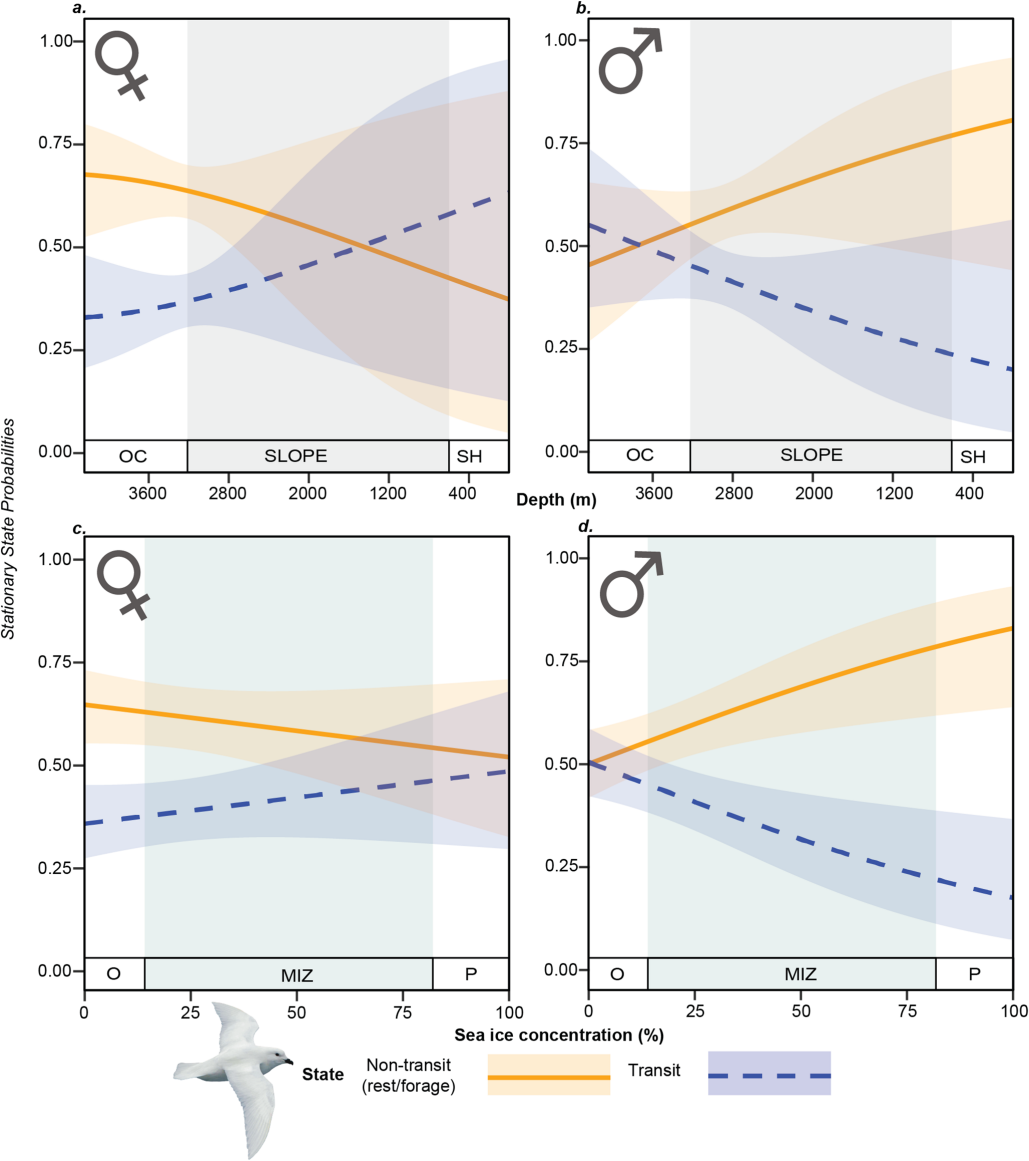
Stationary state probabilities for two Hidden Markov Model-estimated behaviours of snow petrels from two colonies in Dronning Maud Land (orange: rest/forage, purple: travel) for females (***a and c***) and males (***b ***and*** d***) with respect to water depth (***a ***and*** b***) and sea-ice concentration (**c** & **d**). Solid/dashed lines show means and shaded error bars show 95% confidence intervals. For depth (***a and b***), sea-ice concentration was kept at its mean value (19.03%), and for sea-ice concentration (**c **and** d**) water depth was kept at its mean value (2240 m). SLOPE = Continental slope. OC = Oceanic waters. SH = Waters over the continental shelf. P = Pack Ice (sea-ice concentrations > 80%). MIZ = Marginal Ice Zone (sea-ice concentrations < 80% – > 15%). O = Open waters (< 15% sea-ice concentration). Model data compromises 34 trips made by 15 snow petrels. See supplementary tables S1 and S2 for trip counts

Including environmental covariates and their interactions with sex improved the performance of the HMM of behavioural state and the best-supported model included depth and SIC, plus their interactions with sex (Table 3). The second-most supported model included sex, depth and their two-way interaction and had a *Δ*AIC of 1.8. However, as a goal of this study was to characterize both the behavioural response to and use of sea-ice habitats, we retained the model featuring sea-ice concentration as it was more biologically informative. Transition probabilities varied more widely with depth than SIC (Fig. 4). Males were more likely than females to switch to the non-transit state (foraging/resting) over the continental shelf and slope, whereas over oceanic waters the pattern was reversed (Fig. 4). Moreover, stationary state probability distributions (Fig. 5) indicated that as depth increases, females were more likely to be in the non-transit state, however we note that, at shallower depths, the 95% confidence intervals were wide and overlapped substantially. For both sexes, higher probabilities of switching from transit to non-transit occurred in the MIZ and pack ice than in open waters (Fig. 4). Males were more likely to switch to non-transit in ice-covered waters (Fig. 4). Similarly, stationary state probability distributions show that as SIC increased, males were more likely to be in the non-transit state than females (Fig. 5).

**Table 3:** Model selection results based on AIC comparison of the 15 top-ranking candidate 2-state hidden Markov models, ranked by *Δ*AIC from the best-fitting model. SIC = Sea-ice concentration, ice edge = distance to the sea-ice edge^i, ii^

Fixed effects								
Sex	Depth	SIC	Ice Edge	Sex x Depth	Sex x SIC	Sex x Ice Edge	AIC	ΔAIC
✓	✓	✓		✓	✓		79390.5	0.0
✓	✓			✓			79392.2	1.8
✓	✓	✓	✓	✓	✓	✓	79393.9	3.4
✓	✓		✓	✓		✓	79,398	7.5
✓		✓	✓		✓	✓	79400.1	9.6
	✓						79,403	12.5
✓		✓			✓		79403.1	12.7
		✓	✓				79403.5	13
	✓		✓				79403.8	13.3
✓	✓						79404.5	14
	✓	✓	✓				79404.7	14.2
✓			✓				79405.4	14.9
✓			✓			✓	79405.5	14.9
	✓	✓					79,406	15.6
							79410.3	19.9

^i^Model data compromises 34 trips made by 15 snow petrels. See supplementary tables S1 and S2 for trip counts

^ii^Data from both colonies were pooled for these models

## Discussion

In this study, we present a comparative analysis of foraging trip characteristics, habitat use, and behavioural responses to environmental covariates in a sexually dimorphic Antarctic seabird, the snow petrel, breeding at two colonies in East Antarctica.

### Habitat use

Although our study colonies were located ~ 720 km apart in Dronning Maud Land, we found little variation overall in habitat use during early chick rearing, with both colonies using broadly similar foraging habitats in the Lazarev Sea (Svarthamaren) and Kong Haakon VII Sea (Utsteinen). At both colonies, snow petrels had a mean maximum foraging range of 395 ± 32 km during the early chick-rearing period, with one female from Utsteinen reaching 768.3 km from the colony. Previous studies using GLS loggers (Global Location Sensors) in Terre Adélie found a much larger foraging range of 2648 ± 1054 km. However, this figure may reflect trips made during the pre-laying exodus or the movements of failed breeders (Delord et al. 2016). In contrast, GPS-tracking during incubation at Terre Adélie recorded a much smaller maximum range of approximately 150 km (Barbraud et al. 2021). Both the Utsteinen and Svarthamaren colonies are located approx. 200 km inland, whereas the colony in Terre Adélie is along the coastline, which likely explains the difference in maximum foraging range.

Of the environmental covariates in this study, bathymetry was found to have the greatest impact on movement decisions (Figs. 4 and 5; Table 2). Birds utilised waters north of the 500 m isobath, at depths of ~ 3000 m (Fig. 1; Table 2), including waters overlying the shelf break, slope and abyssal plain, and ridges extending offshore, perpendicular to the shelf. Bathymetry was also the main environmental predictor for snow petrels GLS-tracked during the non-breeding period from colonies on Béchervaise and Filla islands in East Antarctica, where birds utilized deep, offshore waters of > 5000 m (Viola et al. 2023). The use of deeper waters than our study may be as a secondary consequence of using the MIZ, which during austral winter is located much further offshore (Delord et al. 2016). In contrast, snow petrels GPS-tracked during incubation from Terre Adélie used shallower waters over the continental shelf (Barbraud et al. 2021). Procellariform seabirds can show intraspecific differences in trip characteristics and habitat use due to regional differences in habitat availability (Cecere et al. 2013), and in our study region the continental shelf is narrower and deeper (Beaman et al. 2011; Eisermann et al. 2024; Hattermann 2018) than that found around the coast of Terre Adélie (Michelot et al. 2020). There, the continental shelf extends approx. 110 km seaward (Colwell et al. 2006), which may provide a greater availability of neritic habitat compared to the narrow continental shelf of Dronning Maud Land, where in places the ice shelves occlude the shelf break (Fig. 1a) (Lowther et al. 2022).

The snow petrel is considered to be an obligate or near-obligate ice-associate (Ainley et al. 2017), and previous GPS-tracking during incubation in Terre Adélie confirmed a high affinity for sea-ice environments (Barbraud et al. 2021). Our results show that both sexes are more likely to switch to putative resting/foraging behaviours in higher sea-ice conditions (Figs. 4 and 5). However, overall, we found use of lower sea-ice conditions than the study at Terre Adélie, with females especially using nominally open waters (< 15% SIC), and males using SIC of ~ 33%. This may have been because our study took place during a period of unusually low SIC for the time of year (Turner et al. 2022). Although seasonal trends in sea-ice extent and concentration in eastern Dronning Maud Land are lower than that at Terre Adélie, and in the former the sea ice may recede all the way to the coast by February (Fig. 1a) (Lowther et al. 2022), both the maximum and minimum sea-ice extents during our study fell below the 30-year median (Fig. 1a). Thus, although the comparatively low regional sea-ice regime may mean that breeding snow petrels at Utsteinen and Svarthamaren either need to forage locally in open water or associated with small patches of sea ice, or travel further afield to forage within the MIZ, the 2022 season may still have led to them encountering lower sea-ice conditions than previously experienced.

Environmental conditions at non-transiting locations did not differ substantially with colony or sex (Fig. 3; Table 2). This may indicate that interactions between these indices, or other processes, not captured by these indices, may also shape snow petrel habitat use. For sea ice, we used the highest resolution data for SIC in the region derived using passive microwave radiometry (Spreen et al. 2008). These comprised daily snapshots of sea-ice concentration per day with a grid cell size of 3.125 km^2^ whereas the temporal resolution of GPS tracking data is much higher (30-minute intervals, accurate to ~ 20 m). As a result, remotely sensed data represent only a snapshot of a dynamic environment, in which SIC and sea-ice type may change continuously in response to wind and currents (Massom and Stammerjohn 2010). Hence, we cannot exclude the possibility that snow petrels apparently foraging in open waters (< 15% SIC) in our study, were in fact foraging in association with ice floes below the threshold of detection for the SIC product used, which has a cell size of 3.125 km (Worby and Comiso 2004; Worby et al. 2008).

### Sex differences

Although both sexes had similar foraging trip durations and distances and allocated the same proportion of time to transiting and non-transiting behaviour, we found sex differences in habitat use and contrasting behavioural changes with respect to depth and sea-ice concentration. Females foraged in deeper, oceanic waters with little to no ice cover, and were also more likely to engage in putative foraging/resting behaviours in areas with these conditions, whereas males used shallower waters over the continental shelf and slope, where SICs were marginally higher. These results are consistent with the previous GPS tracking study of snow petrels in Terre Adélie (Barbraud et al. 2021). However, we caution that these effects were small and, in some cases, only marginally significant, especially for sea-ice concentration.

In seabirds, sex differences in habitat use may arise from several non-mutually exclusive mechanisms. Previous studies have found pronounced sexual size dimorphism in snow petrels; males were 5–10% larger and 20.8% heavier than females (Barbraud and Jouventin 1998; Tveraa and Christensen 2002). Morphological differences, including in wing loading, body mass, and bill structure, can facilitate differential habitat use and spatial distribution. For example, among sexually size-dimorphic giant petrels (*Macronectes* spp.), the larger males dominate terrestrial scavenging of seal carcasses, whereas females more frequently feed offshore, on krill, squid, and fish (Mills et al. 2021; Raya Rey et al. 2012). Among snow petrels, the greater mass of males could provide an advantage for plunge diving at the submerged edges of ice floes, allowing them to exploit prey sources inaccessible to females (Barbraud et al. 1999), or as is the case amongst other procellariforms, allow them to out-compete smaller females for resources (González-Solís et al. 2000). However, among the birds that we tracked, males were only marginally heavier than females and the difference was not significant (Table S2), although this may have been due to our relatively small samples of males. Additionally, the snow petrels at both Svarthamaren and Utsteinen are considered to belong to the smaller of the two described morphs of the species (Bonaparte 1856) and greater variability is observed within the larger morph, *P. nivea major* (Barbraud and Jouventin 1998). Thus, relative size differences and the resultant competitive exclusion between sexes alone may not explain the observed patterns of habitat use between sexes in this study.

Several other hypotheses have been proposed to explain sexual divergence in foraging patterns among seabirds. These include differences between sexes in: nutritional requirements (Strydom et al. 2023; Welcker et al. 2009); reproductive roles (Thaxter et al. 2009); responses to chick condition (Hamer et al. 2006; Quillfeldt et al. 2004); and risk aversion (Congdon and Preker 2004; Elliott et al. 2010). Although we cannot discount any of these as potential causes of the observed habitat partitioning found in this study, we note that, at Terre Adélie during incubation, alongside utilising lower sea-ice concentrations, females forage at a lower trophic level than males (Barbraud et al. 2021).

Foraging at lower trophic levels could indicate a greater reliance on krill over fish by females, possibly because these are more abundant or easily captured than fish in areas where sea ice has recently receded (Delord et al. 2016; Hodum and Hobson 2000; Ridoux and Offredo 1989). The observed habitat divergence in snow petrels in this study may thus reflect sex-dependant dietary niche specialisation, potentially as an adaptation to avoid intersexual competition for resources during the narrow summer breeding period in the Antarctic (Cleasby et al. 2015; Mancini et al. 2013; Phillips et al. 2017). However, it remains unclear whether this is the ultimate cause of the observed sex differences in habitat use or a proximate consequence of habitat segregation for other reasons, and so, precluding further dietary studies in the region during the chick rearing period, this attribution remains speculative. Similarly, although at Terre Adélie no spatial segregation was observed, we note that at one colony in our study, Utsteinen, there is a contrasting westward vs. eastward bias in the 50% core UD for males and females, respectively (Fig. 1b). As the previous study took place during the incubation period whilst ours in early to mid-chick rearing, there is potential that a spatial segregation may occur later in the breeding season.

### Snow petrels in a changing Antarctic sea-ice regime

Our study coincided with a period of record-low sea ice around Antarctica (Descamps et al. 2023; Turner et al. 2022). Satellite records from the preceding three decades indicated a gradual increase in Antarctic sea-ice extent (Parkinson 2019) and the past decade has been marked by extreme variability. Record maxima were observed between 2012 and 2014 followed by a significant negative anomaly beginning in 2015/2016 (Hobbs et al. 2024; Turner et al. 2015) culminating in the then lowest extent on record in 2021/2022 (Turner et al. 2022). However, these overall trends mask substantial regional variability (Eayrs et al. 2021; Parkinson 2019). Our study region spans two sectors of the Southern Ocean, the Atlantic sector (Svarthamaren) and the boundary between the Atlantic and Indian sectors (Utsteinen). In both sectors, minimum sea-ice extent typically occurs in February, with the Indian sector experiencing shorter and less extensive sea-ice cover than the Atlantic sector (Parkinson 2019). However, despite this existing variability (Massom et al. 2013) there is a growing consensus that Antarctic sea ice is undergoing a shift to a new regime, characterized by greater variability (Hobbs et al. 2024).

The diet of snow petrels predominantly consists of sea-ice-associated prey, notably Antarctic silverfish *(Pleuragramma antarctica)* and Antarctic krill *(Euphausia superba)* (Ainley et al. 1984; Fijn 2012; Ridoux and Offredo 1989). The distribution and abundance of these key prey species are closely linked to sea ice, particularly during their juvenile and larval stages (Bottaro et al. 2009; David et al. 2021; Piñones and Fedorov 2016; Vacchi et al. 2004). Consequently, changes in sea-ice dynamics may have bottom-up effects on the foraging success of snow petrels. In our study, we also showed that snow petrels were able to utilise areas with low SIC, but whether this was a response to the unusual conditions of 2022 requires further study, particularly to assess dietary responses to recent periods of reduced sea-ice extent.

## Conclusion

In conclusion, we found use of lower sea-ice concentrations and deeper waters than in previously studies of snow petrels, indicating a potential regional variability in habitat use for the species. We also found evidence of partial sexual segregation of foraging habitat, with females using lower sea-ice concentrations and deeper waters than males, but these effects were small. Our tracking data provide an insight into the foraging behaviour of an archetypal pagophilic seabird during a period of extreme weather and anomalously low sea-ice, conditions which may become more frequent (Turner et al. 2019) as the climatic regime of the southern ocean shifts under the influence of anthropogenic climate change.

## Electronic supplementary material

Below is the link to the electronic supplementary material.

Supplementary Material 1

## Declarations

### Data/Code availability

The tracking datasets analysed during the current study are available in the BirdLife Seabird Tracking Database https://data.seabirdtracking.org/, dataset ID 2274 and will be archived in the UK Polar Data Centre.
