# Supplementary material for: The foraging distribution and habitat use of chick-rearing snow petrels from two colonies in Dronning Maud Land, Antarctica
Source: Mar Biol. 2025 Jun 5;172(7):109. doi: 10.1007/s00227-025-04657-w (PMC12141146; doi:10.1007/s00227-025-04657-w)
Supplement: Supplementary file 1 — Supplementary Material 1 [file 227_2025_4657_MOESM1_ESM.pdf]

Supplementary Information

Article title: The foraging distribution and habitat use of chick-rearing snow petrels from two colonies in Dronning Maud Land, Antarctica

Journal: Marine Biology

Author list: Eleanor Maedhbh Honan[1]*, Ewan D. Wakefield[1], Richard A. Phillips[2], W. James Grecian[1], Stephanie Prince[1], Henri Robert[3], Sébastien Descamps[4], Anna Rix[1,5], A. Rus Hoelzel[5], Erin L. McClymont[1]

[1]Department of Geography, Durham University, Durham, DH1 3LE, United Kingdom.

[2]British Antarctic Survey, Natural Environment Research Council, High Cross, Madingley Road, Cambridge, CB3 0ET, United Kingdom.

[3]International Polar Foundation, Rue des Vétérinaires, 42b/1, 1070 Brussels, Belgium.

[4]Norwegian Polar Institute, Fram Centre, 9296 Tromsø, Norway.

[5]Department of Biosciences, Durham University, Durham, DH1 3LE, United Kingdom.

Corresponding author: **eleanor.m.honan@durham.ac.uk*

Table S1 Initial and estimated parameter values for step length and turning angle for two-state Hidden Markov Models for behavioural state annotation of GPS-tracked snow petrels from two colonies in Dronning Maud Land[i].

State		Step Length	Turning Angle
**State 1**	Range	10 ± 557 – 10000 ± 1671	0 (0.45)
	Optimum	2764.03 ± 2777.05	0 (0.45)
	**Best fit**	2708.5928 ± 2712.573	0 (0.45)
**State 2**	Range	15000 ± 1114 – 30000 ± 2228	0 (0.84)
	Optimum	11281.05 ± 4783.4)	0 (0.83)
	**Best fit**	11247.524 ± 4808.759	0 (0.84)

[i]Model data compromises 34 trips made by 15 snow petrels. See supplementary tables S1 and S2 for trip counts.

[ii]Data from both colonies were pooled for these models.

Table S2 Body mass of male and female snow petrels from two colonies in Dronning Maud Land (Utsteinen = UT, Svarthamaren = SV). Results are presented as mean ± standard deviation (range). We tested for differences between sexes, colonies and each sex within each colony using a Welch Two Sample t-test and report test statistics (t), degrees of freedom (df) and p-value *(p)*. $p < 0.05$ indicates significant differences between groups.

Group	*n.* individuals	Mean ± SD (range)	t	df	*p*
Colony	UT (30)	261.4 ± 26.4 (205, 325)	-1.089	7.05	0.312
	SV (6)	248.3 ± 27.0 (225, 290)			
Sex	M (14)	263.3 ± 19.1 (230, 300)	-0.8	33.472	0.406
	F (21)	256.3 ± 30.9 (205, 325)			
UT Sex	M (13)	266.9 ± 17.5 (240, 300)	-1.1	25.937	0.292
	F (17)	257.2 ± 31.4 (205, 325)			
SV Sex	M (2)	240.0 ± 14.1 (230, 250)	0.7	3.99	0.55
	F (4)	252.5 ± 32.8 (225, 290)			

Table S3 Summary table of trip counts for each individual snow petrel tracked in this study. *Colony* denotes the colony of origin, where SV = Svarthamaren, UT = Utsteinen. *Bird ID* is the unique identifier given to each tracked individual. For sex: F = Female, M = Male, U = Unknown. For breeding stage, IN = Incubation, BG = brood-guard, PB = Post-brood. *n. Trips* denotes the total number of trips obtained from the individual bird. *n. Complete* denotes how many of these trips were not missing portions of any leg (outward commute, at-sea, inward commute) and thus used in generalised linear mixed effect models (GLMMs) for distance and duration metrics. *n. At-sea leg complete* denotes the number of trips where the at-sea portion of the trip was complete and

thus the trip was used in subsequent behavioural state annotation with Hidden Markov Models (HMM) and habitat-use analyses with GLMMs. Where the number of total trips differs from *n. complete* or *n. at-sea complete,* suffixes and footnotes explain the reason as to the trips' exclusion from the models.

Colony	Bird ID	Sex	Breeding Stage	n. Trips	n. Complete	n. At-sea leg complete
SV	SV001	F	BG	1	0[i]	0[ii]
	SV002*	F	BG	1	1	1
	SV002	F	PB	1	1	1
	SV003*	M	BG	1	0[iii]	1
	SV003	M	PB	4	3[iv]	4
	SV004	M	BG	1	1	0[v]
	SV005	F	BG	1	1	1
	SV006	F	PB	1	1	1
UT	UT001	U	IN	1[vii]	0	0
	UT005	F	IN	1[vii]	0	0
	UT007	F	IN	1[vii]	0	0
	UT026	M	PB	4	3 [iv]	4
	UT028	F	PB	1	0[vi]	0[vi]
	UT031	F	PB	6	5[iv]	6
	UT035	F	PB	2	2	2
	UT036	F	PB	1	1	1
	UT040	F	PB	1	0[i]	1
	UT042	F	PB	1	1	1
	UT050	F	PB	1	1	1
	UT053	M	PB	2	1	2
	UT055	M	PB	1	0[i]	1
	UT058	M	PB	5	3[i, iv]	5
	UT062	M	PB	1	0 [iv]	1
	**Total**			40	25	34

[*]*Denotes an individual from whom trips were recovered across multiple stages.*

[i]*Trip missing portion of outward and inward legs due to device error.*

[ii]*Device consistently recorded at intervals > 30 minutes.*

[iii]*Trip missing portion of inward leg due to device error.*

[iv]*Trip missing portion of outward leg due to device error.*

[v]*9 hour gap in location retrieval due to device error.*

[vi]*Trip begins at sea, therefor missing all the outward leg and a portion of the time at sea.*

[vii]*Incubation trip therefor not included in this study.*

Table S4 Table summarizing numbers of GPS-tag deployments on breeding adult snow petrels across two colonies by colony, sex and stage. *Colony* denotes the colony of origin, where SV = Svarthamaren, UT = Utsteinen. For sex: F = Female, M = Male, U = Unknown. For breeding stage, IN = Incubation, BG = Brood-guard, PB = Post-brood. *n. Deployments* represents the total number of tags deployed on individuals during the study. *n. Recoveries* denotes the number of individual birds from whom tracking data were obtained, *n. Trips (Total)* denotes the number of trips in total from the respective sex/stage class. *n. Trips (Trip metric GLMMs)* denotes how many of the total trips were used in generalised linear mixed effect models (GLMMs) to quantify trip distance and duration metrics. *n. Trips (HMM, habitat GLMM)* denotes how many of the total trips were used in the Hidden Markov Models (HMM) for behavioural state annotation and subsequent GLMMs for habitat use.

**Colony**	**Sex (Stage)**	***n.* Deployments**	***n.* Recoveries**	***n.* Trips *(Total)***	***n.* Trips *(Trip metric GLMMs)***	**n. Trips *(HMMs, habitat GLMMs)***
UT	F (IN)	7	2	2	0	0
	M (IN)	2	0	0	0	0
	U (IN)	1	1	1	0	0
	F (PB)	12	7	13	10	12
	M (PB)	12	5	13	7	13
Colony total		34	15	29	17	25
SV	F (BG)	4	3*	3	2	2
	M (BG)	0	2*	2	2	2
	F (PB)	2	2	2	1	1
	M (PB)	0	1	4	3	4
Colony total		6	6	11	8	9
Study total		*40*	*21*	*40*	*25*	*34*

Denotes an individual from whom trips were recovered across multiple stages.

Table S5 Fixed effects in a generalised linear mixed-effect model testing the influence of sex and colony on the portion of time per trip spent in the non-transit state assigned using a two-state Hidden Markov Model on GPS-tracked snow petrels from two colonies in Dronning Maud Land, Antarctica.

**Response (*error family, link function)***	**Covariate**	**Estimate**	**SE**	***z***	***p***
Proportion of time in non-transit state *(Beta-regression, logit)*	Intercept (females, SV)	-0.121	0.105	-1.150	0.250
	Sex (males)	0.135	0.094	1.430	0.153
	Colony (UT)	0.102	0.107	0.953	0.341

Table S6 Fixed effects in Generalised linear mixed-effects models of maximum latitude (i.e. northerly extent), minimum longitude (westerly extent) and maximum longitude (easterly extent) at non-transiting locations used by snow petrels GPS-tracked from two colonies in Dronning Maud Land, Antarctica[i].

Response (error family, link function)	**Covariate**	**Estimate**	**SE**	***z***	***p***
Max. Latitude - Utsteinen	Intercept (females)	-68.787	0.266	-258.97	<0.001
(Gaussian, Identity)	Sex (males)	-0.228	0.419	-0.54	0.586
Min. Longitude - Utsteinen	Intercept (females)	20.879	0.613	34.05	<0.001
(Gaussian, Identity)	Sex (males)	0.357	0.946	0.38	0.706
Max. Longitude - Utsteinen	Intercept (females,)	26.367	0.711	37.08	<0.001
(Gaussian, Identity)	Sex (males)	1.185	1.165	1.02	0.309
Max. Latitude - Svarthamaren	Intercept (females)	-69.298	0.066	-1047.1	<0.001
(Gaussian, Identity)	Sex (males)	0.066	0.093	0.7	0.481
Min. Longitude - Svarthamaren	Intercept (females)	-3.451	0.264	-14	<0.001
(Gaussian, Identity)	Sex (males)	-0.054	0.349	-0.16	0.877
Max. Longitude - Svarthamaren	Intercept (females)	6.6158	0.575	11.5	<0.001
(Gaussian, Identity)	Sex (males)	-0.608	0.814	-0.75	0.455

[i]Models were run separately for each colony.
